# Heat Transfer and Entropy in a Vertical Porous Plate Subjected to Suction Velocity and MHD

**DOI:** 10.3390/e23081069

**Published:** 2021-08-18

**Authors:** N. Ameer Ahammad, Irfan Anjum Badruddin, Sarfaraz Kamangar, H.M.T. Khaleed, C. Ahamed Saleel, Teuku Meurah Indra Mahlia

**Affiliations:** 1 Department of Mathematics, Faculty of Science, University of Tabuk, Tabuk 71491, Saudi Arabia; anaudalur@ut.edu.sa; 2 Mechanical Engineering Department, College of Engineering, King Khalid University, Abha 61421, Saudi Arabia; sarfaraz.kamangar@gmail.com (S.K.); ahamedsaleel@gmail.com (C.A.S.); 3 Department of Mechanical Engineering, Faculty of Engineering, Islamic University of Madinah, Madinah Munawwarra 42351, Saudi Arabia; khalid_tan@yahoo.com; 4 Centre of Green Technology, Faculty of Engineering and Information Technology, University of Technology, Sydney, NSW 2007, Australia

**Keywords:** porous medium, vertical plate, entropy, MHD

## Abstract

This article presents an investigation of heat transfer in a porous medium adjacent to a vertical plate. The porous medium is subjected to a magnetohydrodynamic effect and suction velocity. The governing equations are nondepersonalized and converted into ordinary differential equations. The resulting equations are solved with the help of the finite difference method. The impact of various parameters, such as the Prandtl number, Grashof number, permeability parameter, radiation parameter, Eckert number, viscous dissipation parameter, and magnetic parameter, on fluid flow characteristics inside the porous medium is discussed. Entropy generation in the medium is analyzed with respect to various parameters, including the Brinkman number and Reynolds number. It is noted that the velocity profile decreases in magnitude with respect to the Prandtl number, but increases with the radiation parameter. The Eckert number has a marginal effect on the velocity profile. An increased radiation effect leads to a reduced thermal gradient at the hot surface.

## 1. Introduction

Porous media are used in several applications involving a wide range of scientific and engineering fields. These diverse applications have motivated many researchers to explore the characteristics of porous media when subjected to various phenomena. Certain phenomena, such as heat transfer and fluid flow, have resulted in many scientific research articles explaining the meticulous details pertaining to heat transfer inside porous media. This number of articles is justified due to the varied applications where porous media play an important role. For example, foams that have several engineering applications are a form of porous media. Iasiello et al. [1] studied open-cell metal foams and their characteristics with respect to anisotropic heat transfer. Iasiello et al. [2] noted that performance criteria increased by 42% in foams, accounting for the variable porosity and cell size. It is known that metal foam has the capability to significantly enhance heat transfer because of thermal resistance reduction in heat-carrying fluid [3]. Porous foams have been successfully investigated in energy storage applications as well [4,5]. They have also been used in heat exchangers [6] and solar receiver applications [7]. A porous medium adjacent to a vertical plate is one of the classical fields of study that has attracted researchers over the years. Generally, heat transfer is studied when the vertical plate is heated to a higher temperature, which allows the heat to be transported into the porous medium due to conduction and convection caused by moving fluid contained in the medium. It is noted that temperature and velocity decay as the fluid moves deep inside the porous medium, perpendicular to the applied heat source. For instance, the velocity increases near the vertical plate, and then declines in a perpendicular direction to the plate, as observed by Raptis [8]. In this case, the analytical solution for the temperature and velocity was deduced from the fundamental equations. Huang [9] analyzed internal heat generation due to fluid flow, following non-Newtonian behavior and subjected to thermal radiation, considering the Soret and Dufour effects along with the porous vertical plate. It was noted that the power-law index of non-Newtonian fluid has a certain impact on temperature profile, and the increased power-law index tends to decrease the temperature gradient at the hot surface. In the case of mass transfer coupled with heat transfer, the buoyancy ratio has a positive effect, which increases the heat and mass transfer rate from the surface to the medium [10]. The variable viscosity can also have some impact on the heat and mass transferred through the porous media. It was noted that the velocity decreased when the viscosity parameter increased [11], which can be attributed to the strong dependence of fluid flow on viscosity. The study of porous media adjacent to the vertical plate has inspired the analysis of different types of fluids. For instance, Khan et al. [12] examined the case of Casson fluid flowing in a porous medium bounded by a vertical plate with other parameters, such as chemical reaction, heat generation, and magnetic field. They relied on the Laplace transform to obtain the exact solution to the transient problem they investigated. It was noted that the velocity profile, concentration, and temperature increased with time. It was also noted that for an oscillating plate, the permeability of the porous medium should be decreased in order to reduce the velocity profile. However, it was noted by Rajakumar et al. [13] that the permeability increases the velocity profile with respect to a vertical plate, near the vicinity of the plate, and it does not have any significant effect deep in the porous medium. It was also reported that the increase in the chemical reaction parameter increases the velocity in the porous medium. In the case of spanwise fluctuating heat and mass transfer due to transient free convection across a vertical porous plate, the concentration is reduced when the Reynolds number increases. It was observed that at the initial stage the mass transfer of fluid was high and then it followed into the boundary layer pattern [14]. The motion of a vertical plate across porous media due to non-Newtonian fluid was analyzed by Nabil et al. [15]. The fluid was assumed to follow the Eyring-Powell model with the plate moving in an opposite direction to that of gravity. An increased Grashof number was found to also increase the velocity of the fluid in the medium. They too found that an increase in chemical reaction increases the velocity profile. Radiation can also have a significant effect on heat transfer with regard to the porous medium. The radiation term is generally introduced using the Rosseland approximation theory, and further simplified by adopting Taylor’s series [16,17,18,19]. Radiation is found to enhance the heat transfer rate from the heating source to the adjacent medium, as reported in many studies [20,21,22,23,24]. The increase in heat transfer is consistent even when radiation is coupled with other phenomena, such as mass transfer, magnetic effect, etc.

In recent years, interest in porous media has evolved to include recent trends such as entropy generation. This trend has gained momentum particularly due to the desire to design equipment that can perform by consuming less energy while also reducing waste. Many efforts have been made to understand entropy generation associated with various processes. For instance, Sayed and Abdel-Wahed [25] investigated magnetohydrodynamics and entropy over a moving permeable plate. They concluded that due to an increasing vortex viscosity, the Newtonian fluids have a lesser entropy generation than the non-Newtonian fluids. The presence of nanoparticles in the fluid has marked a difference in entropy generation within any porous domain. It was also shown that the velocity profile declines due to an increase in the buoyancy ratio parameter, and in addition, increasing the temperature difference parameter leads to a reduction in entropy generation number [26]. In another study by Shit and Mandal [27], it was found that the temperature increases due to an increase in the thermophoretic parameter. It was also observed that the Bejan number increases as a result of increased radiation near the heated plate, but when away from the plate, it decreases. The heat transfer rate can be reduced when the Hartman number is increased, which also reduces the thermal entropy generation [28]. Some other important studies with respect to entropy generation can be found in [29,30,31,32,33,34,35].

Entropy generation is an unwanted element that must be reduced to the greatest possible extent to enhance the performance of devices with respect to their energy consumption. Owing to its numerous applications, it is of paramount importance to understand the entropy characteristics of a porous medium with MHD adjacent to a hot vertical plate. This article is novel for its study of entropy generation in a porous medium adjacent to a vertical plate subjected to suction velocity, and magneto hydrodynamics have not been previously reported to the best of our knowledge. Thus, the current work is an attempt in that particular direction.

## 2. Methodology

**Figure 1:**
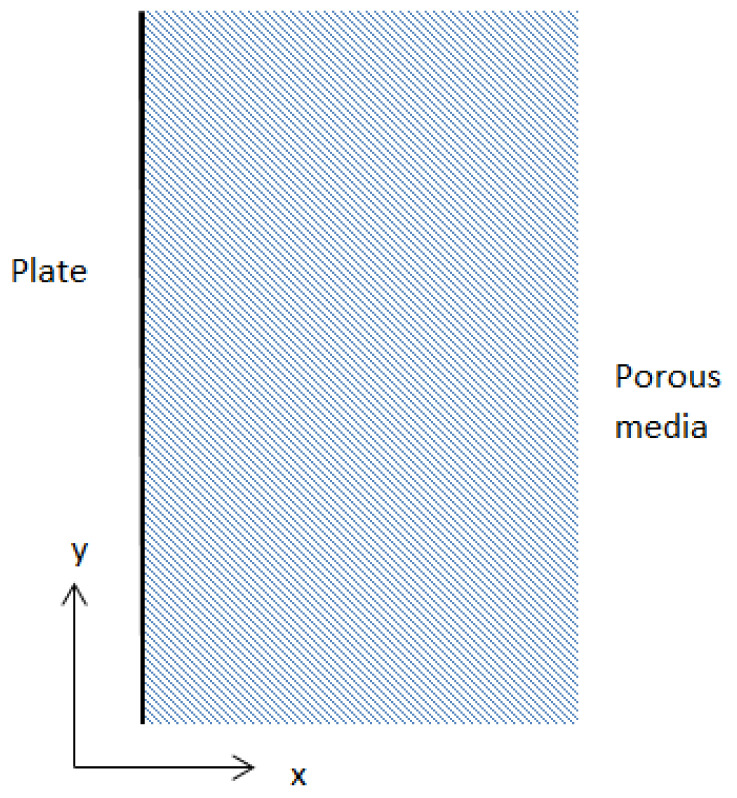
Schematic of the problem.

The problem under investigation belongs to a steady flow of viscous, incompressible fluid across a porous medium bounded by a vertical plate, as shown in Figure 1. The fluid is assumed to be non-scattering, grey-emitting, and radiation-absorbing. The normal *x* and *y* directions are considered along with the horizontal and vertical directions. The properties of the fluid are considered to be constant, apart from the density that allows the natural convection of fluid to take place. The temperature variation is considered only in the body-force term. The governing equations can be given as:

Continuity equation:

(1) $$\frac{\partial u}{\partial x}=0$$

Momentum equation:

(2) $$u\frac{\partial v}{\partial x}=g\beta_{T}\left(T-T_{\infty }\right)+\nu \frac{\partial^{2}v}{\partial x^{2}}-\frac{\sigma B^{2}{}_{o}}{\rho }v-\frac{\nu }{K}v-\frac{c_{F}}{K^{1/2}}v^{2}$$

Energy equation:

(3) $$u\frac{\partial T}{\partial x}=\frac{k}{\rho C_{p}}\frac{\partial^{2}T}{\partial x^{2}}-\frac{1}{\rho C_{p}}\frac{\partial q_{r}}{\partial x}+\frac{\sigma B^{2}{}_{o}}{\rho C_{p}}v^{2}+\frac{\mu }{KC_{p}}v^{2}$$

The continuity (1) leads to:

(4) $$u=-u_{o}$$

where u*_o_* is the constant suction velocity normal to the plate.

Using the Rosseland hypothesis [36,37] as:

(5) $$q_{r}=-\frac{4n^{2}\sigma }{3\beta_{R}}\frac{\partial T^{4}}{\partial x}$$

$T^{4}$ can be expressed as:

(6) $$T^{4}\approx 4T_{\infty }{}^{3}T-3T_{\infty }{}^{4}$$

The Rosseland approximation is valid for an optically thick medium. Excluding the higher-order terms of Taylor’s series expansion would result in a negligible error in Equation (6)

In view of Equations (4)–(6), the Equations (2) and (3) can be shown to have the form as:

(7) $$-u_{0}\frac{\partial v}{\partial x}=g\beta (T-T_{\infty })+\nu \frac{\partial^{2}v}{\partial x^{2}}-\frac{\sigma B^{2}{}_{o}}{\rho }v-\frac{\nu }{K^{\prime }}v-\frac{c_{F}}{K^{\prime }{}^{1/2}}v^{2}$$

(8) $$-u_{o}\frac{\partial T}{\partial x}=\frac{k}{\rho C_{p}}\frac{\partial^{2}T}{\partial x^{2}}+\frac{16n^{2}\sigma^{*}T^{3}{}_{\infty }}{3\rho C_{p}k^{*}}\frac{\partial^{2}T}{\partial x^{2}}+\frac{\sigma B^{2}{}_{o}}{\rho C_{p}}v^{2}+\frac{\mu }{K^{\prime }C_{p}}v^{2}$$

The related boundary conditions of present problem are given as:

(9) $$v=0\;T^{\prime }=T^{\prime }{}_{w}\;\;\mathrm{at}\;x=0$$

(10) $$v\to 0,\;T^{\prime }\to T^{\prime }{}_{\infty }\;\mathrm{as}\;x\to \infty ,$$

where $T^{\prime }{}_{w}$ is the temperature of the plate.

The following non-dimensional parameters are used

(11) U=vuo  X=xuoνθ=T−T∞Tw−T∞Pr=ρν Cpk   G=ν gβ(Tw−T∞)uo3K=uo2ν2K′  N= 4σn2 T∞3βRksε=αμΔTK′ρCp M=σB2oνuo2ρ   E=uo2CpΔTRe=uoLν  Br=μ.uo2k.ΔT2Tp=ΔTT∞

Substituting the above non-dimensional parameters into Equation (7) and (8) yields:

(12) $$\frac{d^{2}U}{dX^{2}}+\frac{dU}{dX}+G.\theta -M.U-\frac{U}{K}-\frac{c_{F}U^{2}}{K^{1/2}}=0$$

(13) $$(1+4N/3)\frac{d^{2}\theta }{dX^{2}}+\mathrm{Pr}\frac{d\theta }{dX}+M.\mathrm{Pr}.E.U^{2}+{\mathrm{Pr}}^{2}.D..U^{2}=0$$

The boundary conditions take the form

(14) U=0  θ=1  at  X=0

(15) U→0, θ=0  as X→∞

### 2.1. Entropy Generation

The entropy generated inside the porous medium due to various factors can be described as [38]

(16) $$S_{g}=\frac{k}{T_{\infty }}\left[1+\frac{16n^{2}\sigma^{*}T^{3}{}_{\infty }}{3kk^{*}}\right]{\left(\frac{\partial T}{\partial x^{}}\right)}^{2}+\frac{\mu }{T_{\infty }}\left[{\left(\frac{\partial v}{\partial x^{}}\right)}^{2}+\frac{v^{2}}{K^{\prime }}\right]+\frac{\sigma B^{2}{}_{o}}{T_{\infty }}v^{2}$$

Equation (16) includes the entropy generation due to conductive and radiative heat transfer, viscous effects and the magnetic effect

The non-dimensional form of entropy can be deduced as

(17) SGEN=Re2(1+4N/3)θ2+Br.Re2.1Tp∂U∂X2+1KU2+M.Br.Re.U2

The first term in Equation (17) represents the entropy generation due to heat transfer; the second term highlights the fluid flow entropy; and the last term shows the entropy generated due to an applied magnetic field, which can be individually shown as

(18) Heat transfer related entropy    ST=Re2(1+4N/3)θ2

(19) Fluid flow−related entropy   SF=Br.Re2.1Tp∂U∂X2+1KU2

(20) Magnetic field related entropy  SM=M.Br.Re.U2

### 2.2. Numerical Scheme

**Figure 2:**
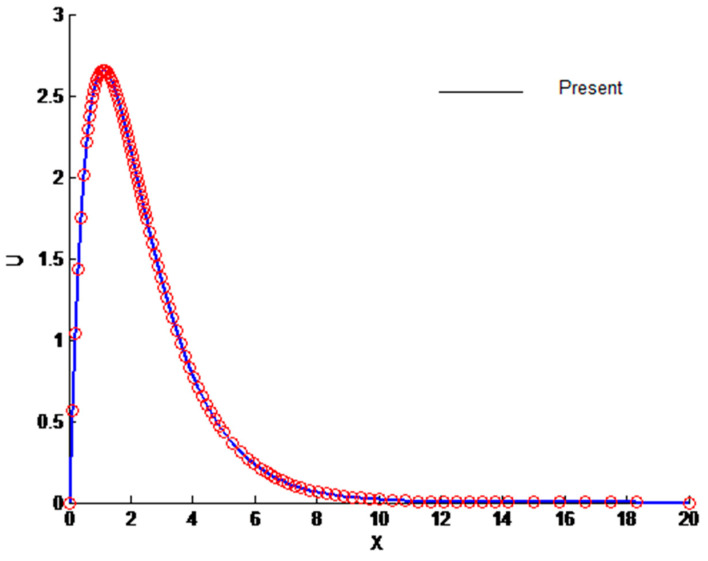
Comparison of velocity profile.

**Figure 3:**
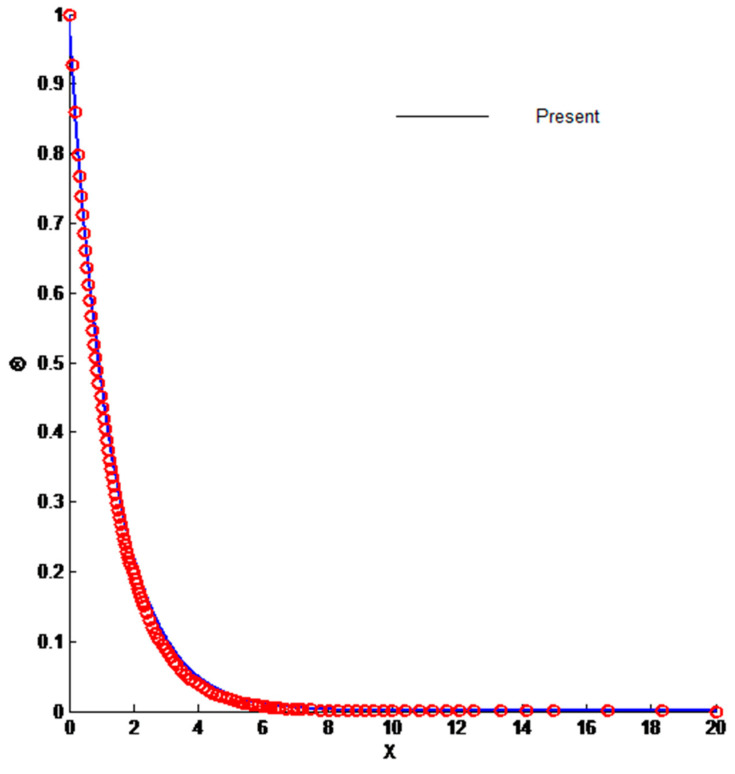
Comparison of temperature profile.

The physical insight of the problem under investigation can be obtained by solving Equations (12), (13) and (17). These equations are solved numerically by the finite difference method. The outcome of the solution is the velocity and temperature profiles, along with the entropy generation. These equations are further simplified by converting them into first-order ordinary differential equations that make them easier to solve. The domain is divided into 1000 points in order to obtain the desired accuracy of 10^−5^. The results are compared to available data to ascertain the accuracy of the current methodology. The comparison is shown in Figure 2 and Figure 3, which clearly demonstrates the accuracy of the present method.

## 3. Results and Discussion

The investigation was carried out with respect to various parameters involved in the governing equations, including the Prandtl number, radiation parameter, permeability parameter, Grashof number, viscous dissipation parameter, magnetic parameter, Eckert number, Reynolds number, and Brinkman number. The results are plotted in terms of velocity, the temperature profile in the porous domain, as well as entropy generation with respect to heat transfer, fluid flow, and magnetic effects.

### 3.1. Velocity and Temperature Profile

**Figure 4:**
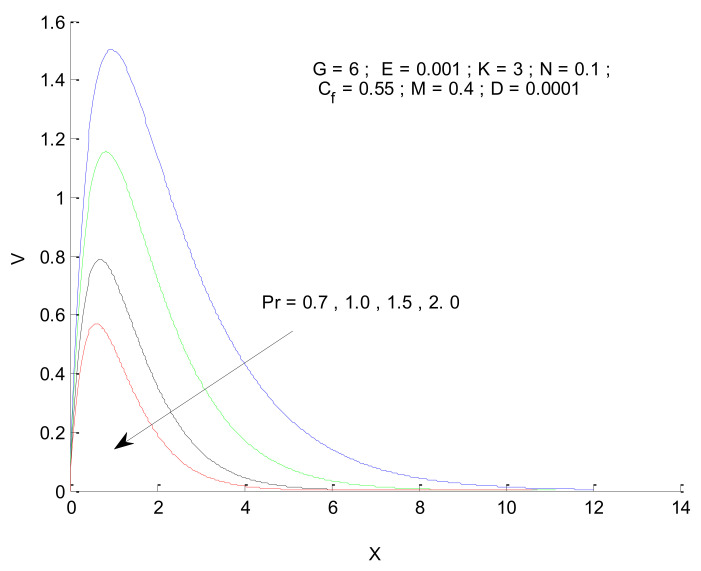
Effect of Prandtl number on velocity profile.

**Figure 5:**
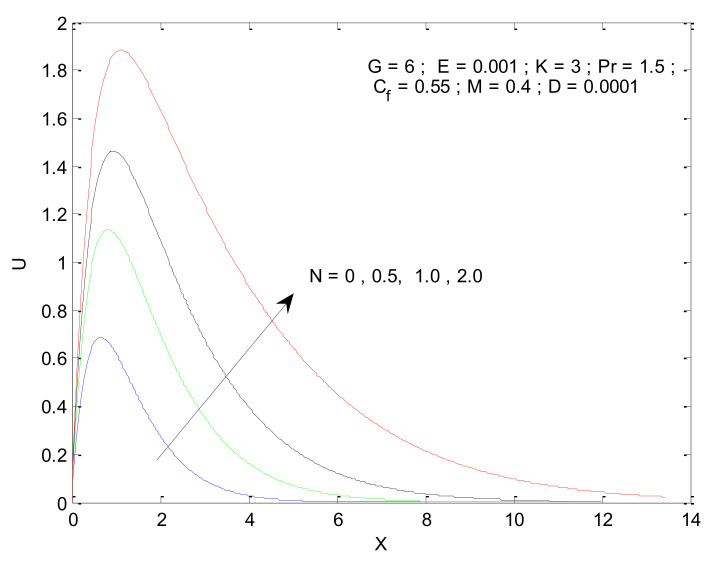
Effect of radiation parameter on velocity profile.

**Figure 6:**
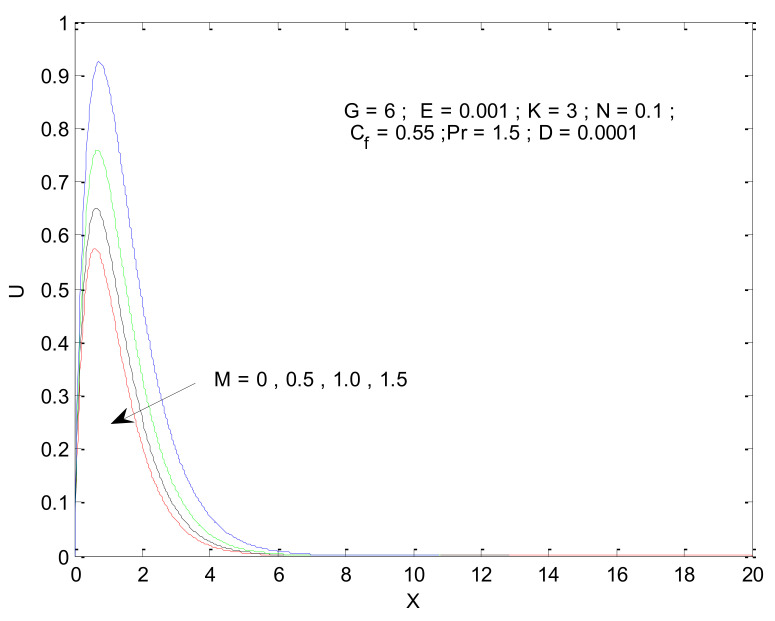
Effect of magnetic parameter on velocity profile.

**Figure 7:**
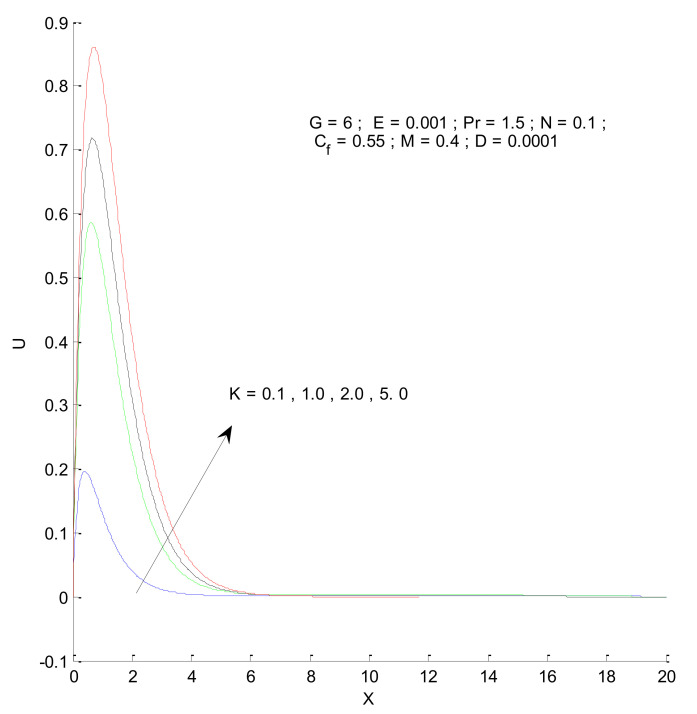
Effect of permeability parameter on velocity profile.

**Figure 8:**
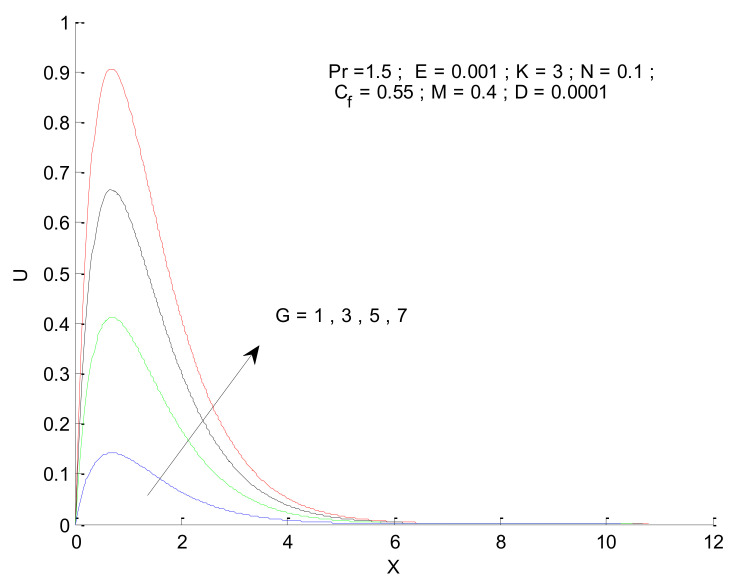
Effect of Grashof number on velocity profile.

**Figure 9:**
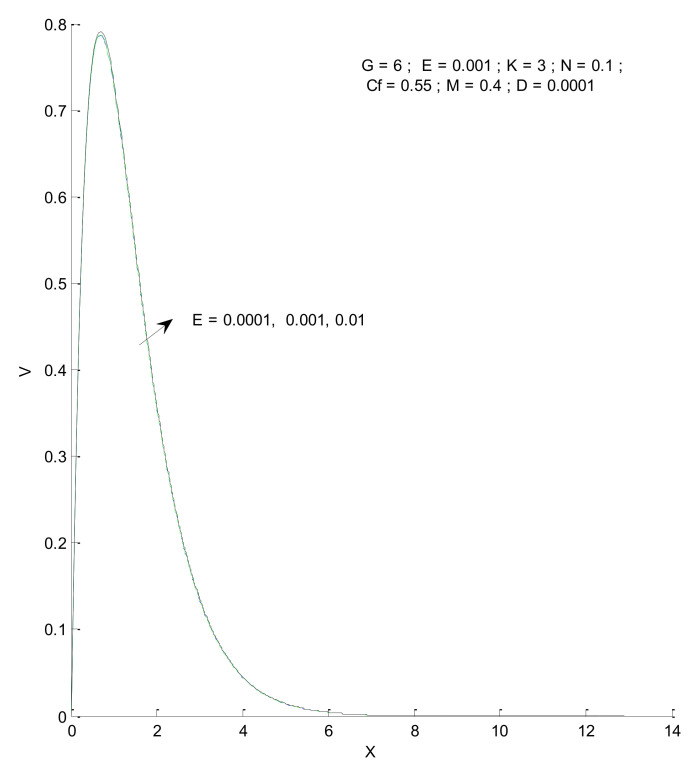
Effect of Eckert number on velocity profile.

**Figure 10:**
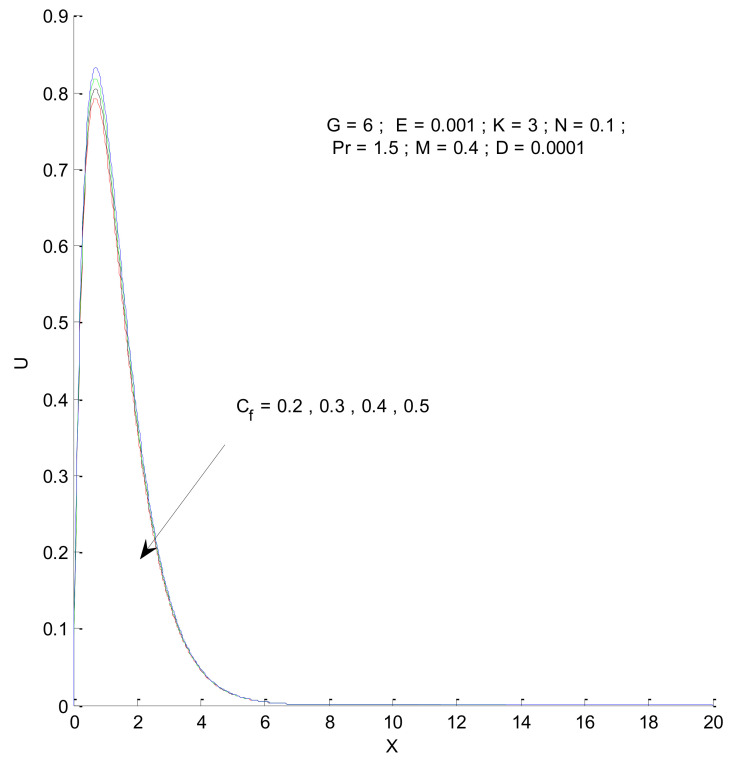
Effect of form-drag on velocity profile.

**Figure 11:**
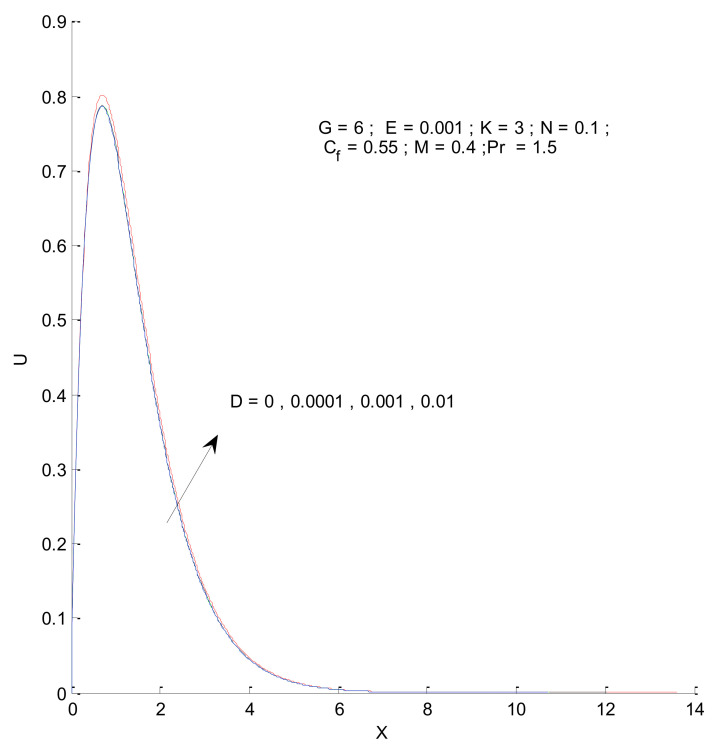
Effect of viscous dissipation parameter on velocity profile.

Figure 4, Figure 5, Figure 6, Figure 7, Figure 8, Figure 9, Figure 10 and Figure 11 show the non-dimensional velocity distribution in the medium with respect to different parameters. The general trend in the velocity profile is to initially increase until a certain distance near the vertical plate and then decline as it moves deep into the porous medium. The sharp increase in velocity near the plate is associated with the higher amount of thermal energy available for fluid due to a heated surface. This, in turn, sets the fluid into higher motion. As the fluid moves away from the plate, it losses kinetic energy due to friction, which then leads to a reduction in velocity. Figure 4 shows that the velocity profile is a strong function of the Prandtl number. The velocity profile squeezes when the Prandtl number is increased, as illustrated by Figure 4. The increase in Prandtl number (Pr) is associated with an increase in viscosity, which hinders the fluid moment, resulting in the reduction in the velocity profile. The impact of Pr on the velocity profile is to decrease the maximum velocity as well as reduce its penetration in the porous medium along the horizontal direction. Figure 5 illustrates the impact of the radiation parameter (N) on the velocity profile. It is found that the velocity increases due to an increase in radiation parameter, which in turn reflects the increased radiation. The increased radiation provides extra thermal energy to the fluid, which is used to increase the fluid’s velocity. The penetration of the velocity into the porous medium increases due to an increase in radiation parameters.

Figure 6 shows the impact of the magnetic field on velocity distribution in the porous medium. Higher values of the magnetic parameter (M) tend to reduce the velocity in the domain. The presence of a magnetic field has a negative impact on the velocity, which decreases owing to an increase in M. The rise and fall of velocity are sharp due to the impact of magnetic parameters compared to the other parameters, as discussed with respect to Prandtl number and the Radiation parameter (Figure 4 and Figure 5). The impact of varying the permeability parameter is shown in Figure 7. The velocity is found to increase with the permeability of the porous medium. It is seen that the rate of decline in velocity after reaching the peak value is higher when the permeability parameter is increased. Figure 8 demonstrates the impact of the Grashof number (G) on velocity distribution. The velocity increases with an increase in G, which may be attributed to the increased buoyancy force when the Grashof number increases. The increased buoyancy force allows the fluid to have higher momentum. The velocity is found to reach a minimum value around X = 6 for all tested values of G. The effects of the Eckert number (E), form-drag (C_f_), and viscous dissipation parameter (D) are shown in Figure 9, Figure 10 and Figure 11. These three parameters have a negligible impact on the velocity profile in the porous medium.

**Figure 12:**
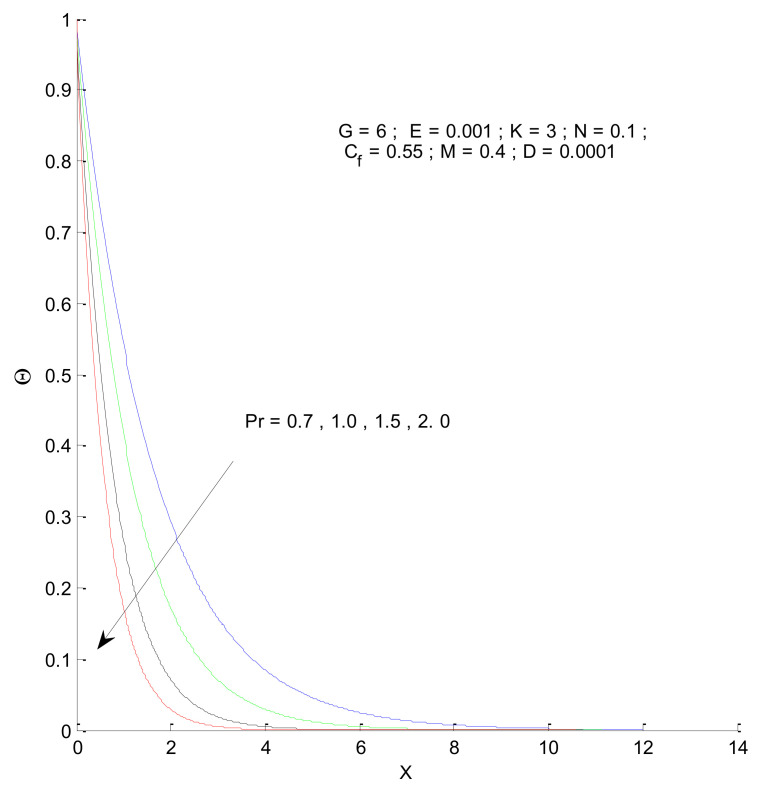
Effect of Prandtl number on temperature profile.

**Figure 13:**
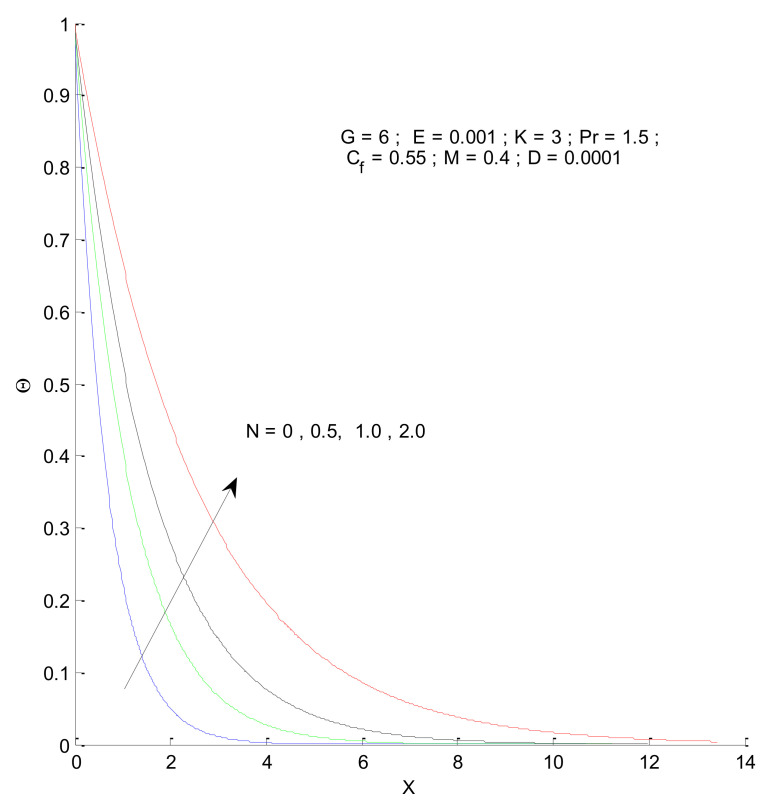
Effect of radiation parameter on temperature profile.

This section describes the temperature variations in the porous domain due to various parameters. Figure 12 shows the temperature distribution when the Prandtl number varies. The thermal boundary layer is thinner for a higher Prandtl number, indicating that the heat transfer should increase from the vertical plate to the porous medium. The temperature variations approach that of the outside condition at a faster rate for the higher value of Pr. The thermal boundary layer expands due to the presence of radiation, as shown in Figure 13. This is because the conduction effect, due to the combination of thermal conduction and radiation, increases with the increase in radiation parameter (N), which can be seen from the constant 1 + 4N/3, in the first term of Equation (13). When there is no radiation corresponding to the value N = 0, the convective effect dominates with a thinner boundary layer.

### 3.2. Entropy Generation

Entropy is a term that describes the disorder or usefulness of energy in any process. Thus, the entropy presents an overview of how efficient a process is by reducing its irreversibility. The lesser the irreversibility, the better the system performance. Entropy can be generated due to various factors such as heat transfer, fluid flow, and magnetic effect. This section is devoted to understanding the different types of entropy generation associated with the current problem. The individual entropy is listed for three terms arising of Equation (17), which are thermal entropy (ST), fluid flow entropy (SF), and magnetic-field-related entropy (SM).

**Figure 14:**
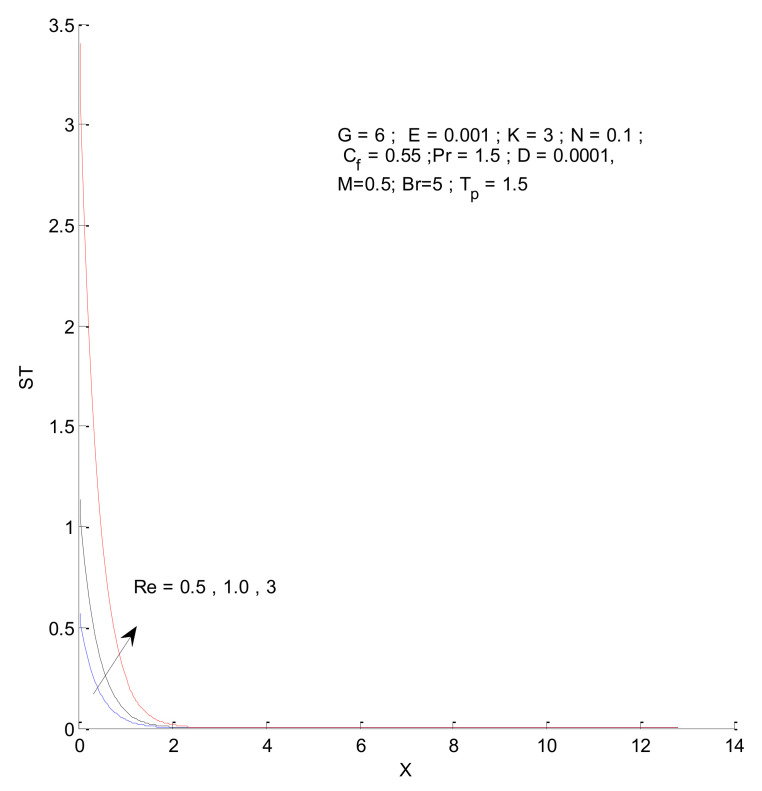
Thermal entropy change with respect to Re.

**Figure 15:**
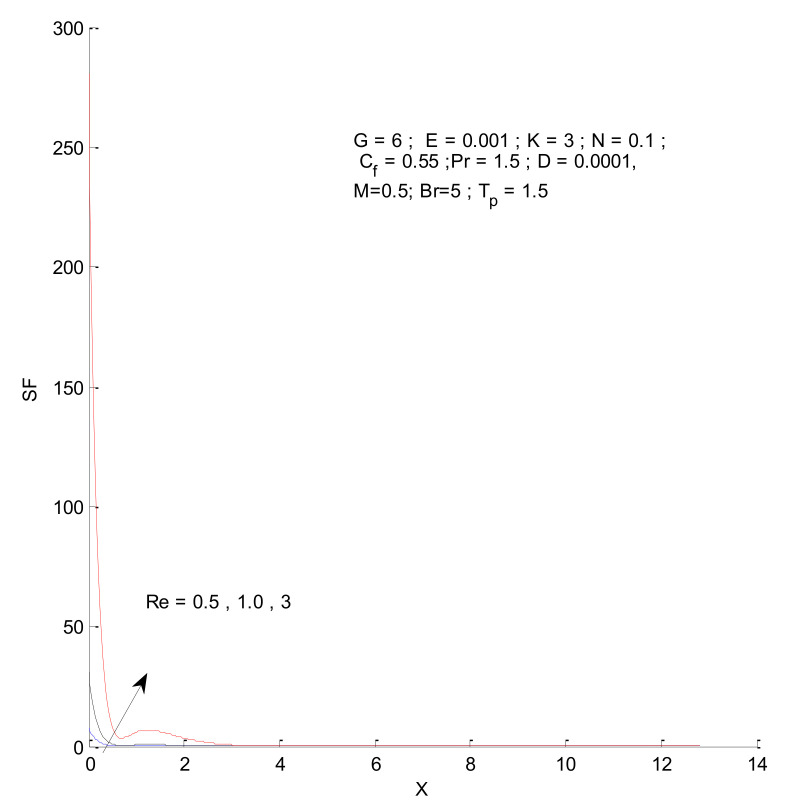
Fluid entropy change with respect to Re.

**Figure 16:**
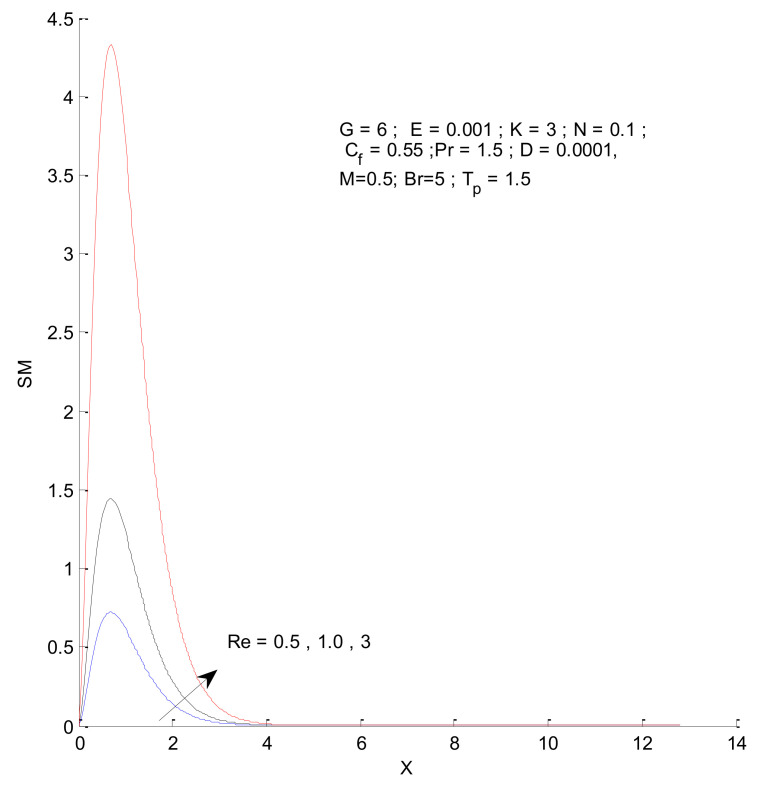
Magnetic entropy change with respect to Re.

**Figure 17:**
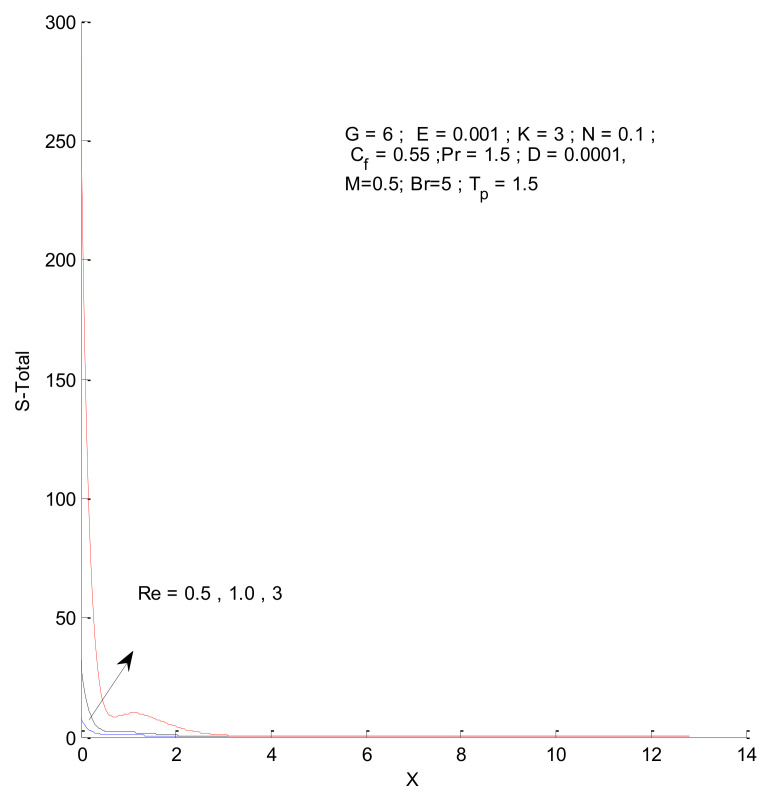
Total entropy change with respect to Re.

Figure 14, Figure 15, Figure 16 and Figure 17 show the entropy variation in ST, SF, SM, and S-Total with respect to changes in the Reynolds number. It is found that the entropy generation increases with an increase in the Reynolds number. The Reynolds number represents the ratio of inertia to viscous force. The increased inertia force leads to higher entropy for ST, SF, and SM, as indicated by Figure 14, Figure 15 and Figure 16. The thermal and fluid entropy is higher near the vertical plate, whereas the magnetic entropy initially increases and then decreases. The total entropy, which is the summation of all individual entropies, is dominated by the fluid flow entropy.

**Figure 18:**
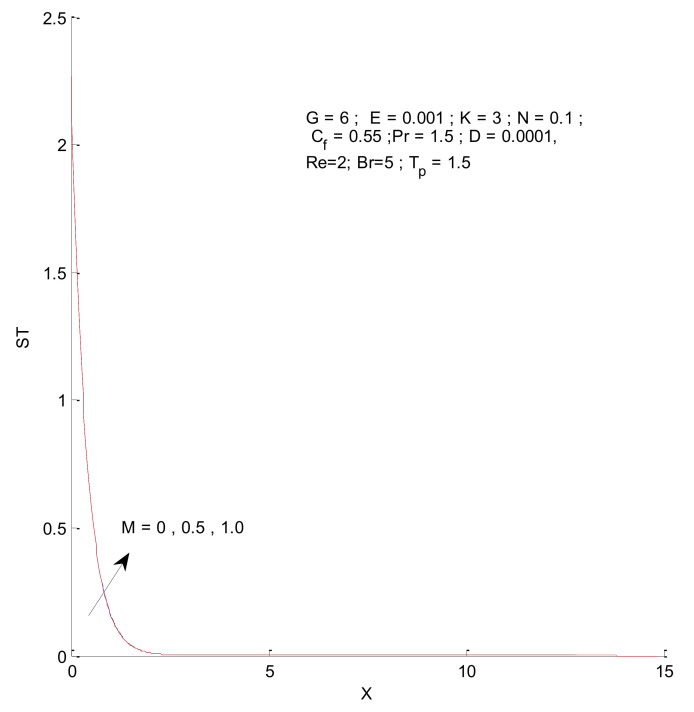
Thermal entropy change with respect to M.

**Figure 19:**
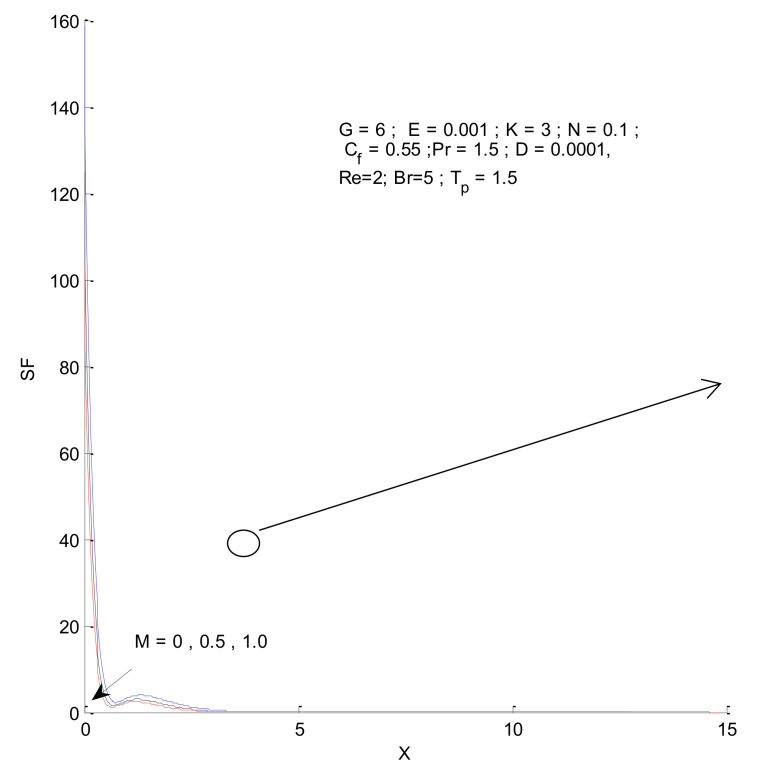
Fluid entropy change with respect to M.

**Figure 20:**
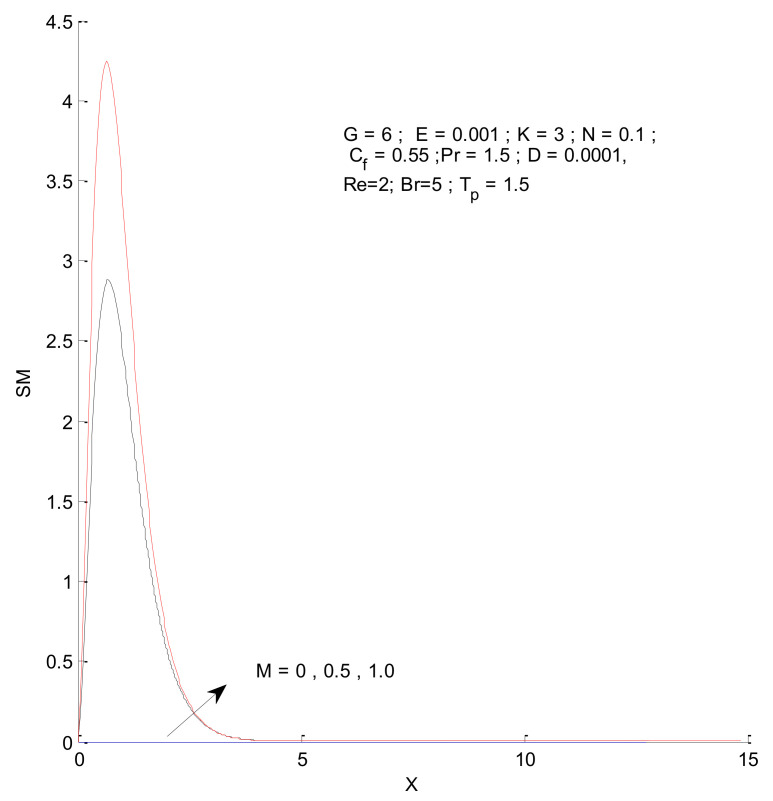
Magnetic entropy change with respect to M.

**Figure 21:**
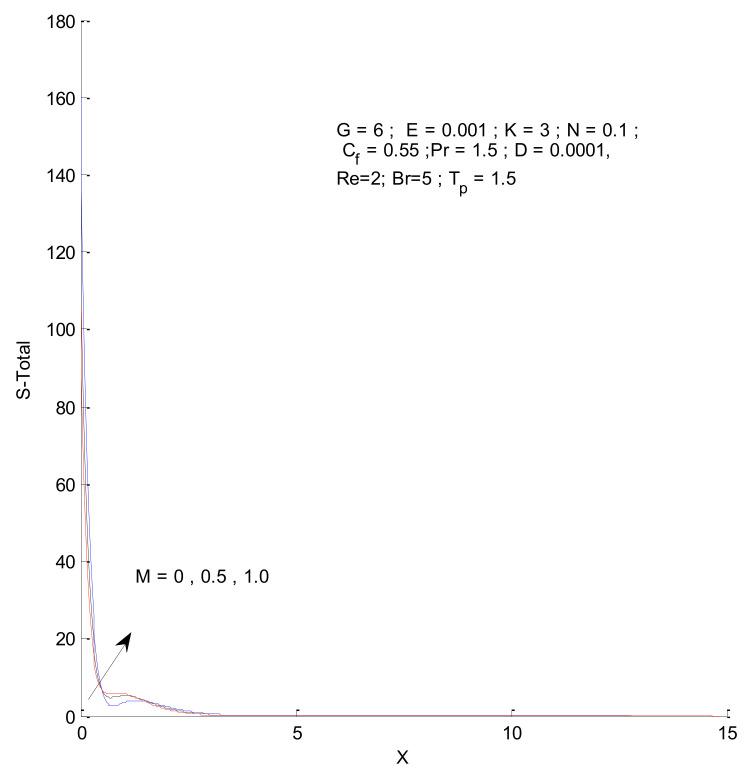
Total entropy change with respect to M.

**Figure 22:**
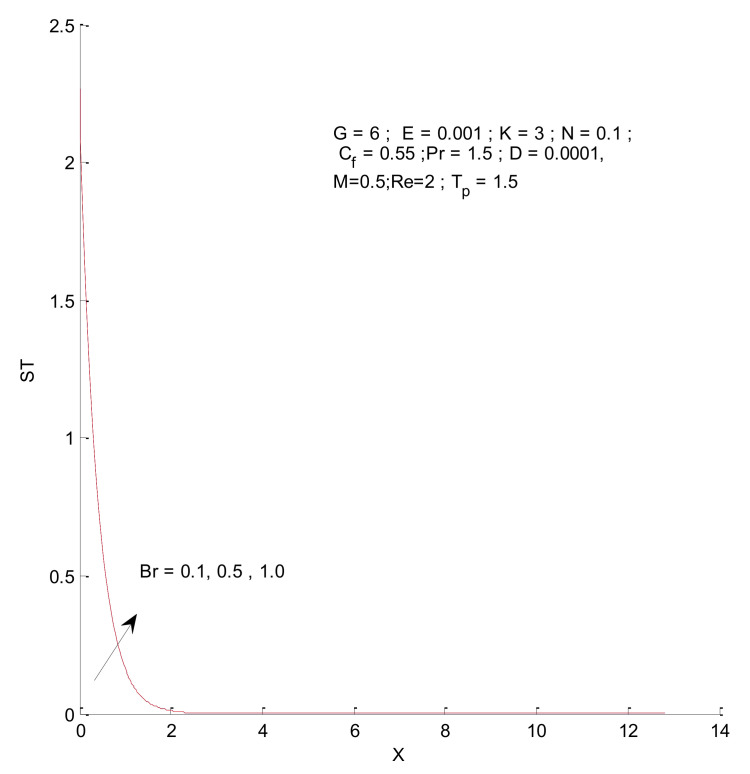
Thermal entropy change with respect to Br.

**Figure 23:**
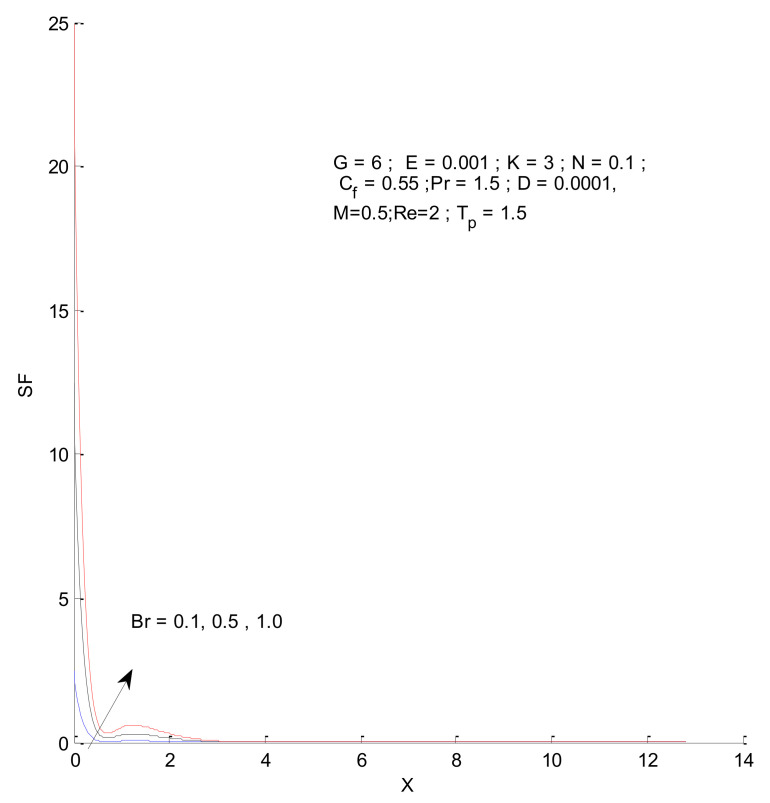
Fluid entropy change with respect to Br.

**Figure 24:**
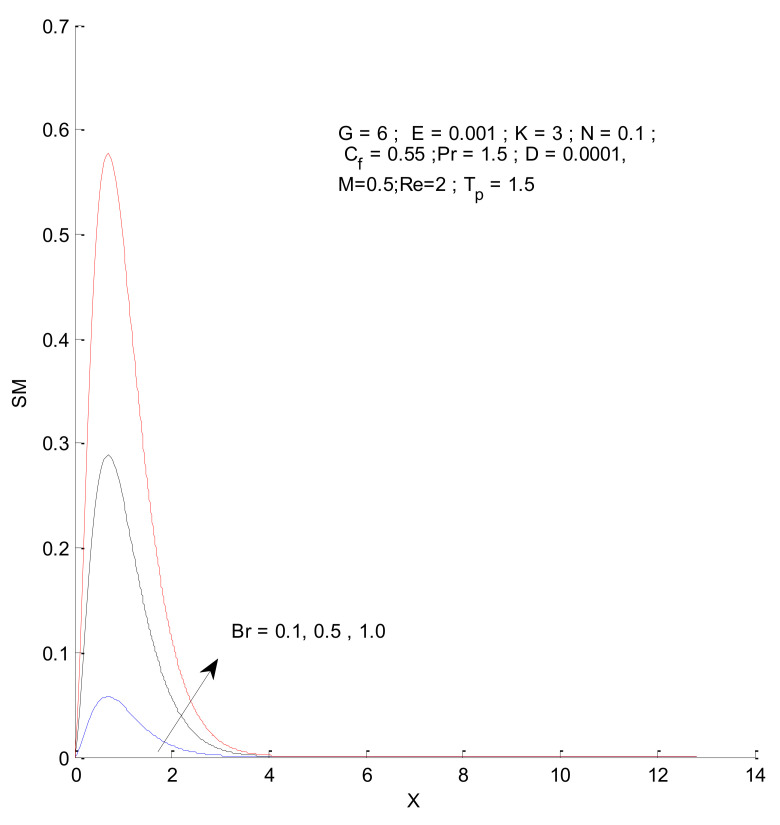
Magnetic entropy change with respect to Br.

**Figure 25:**
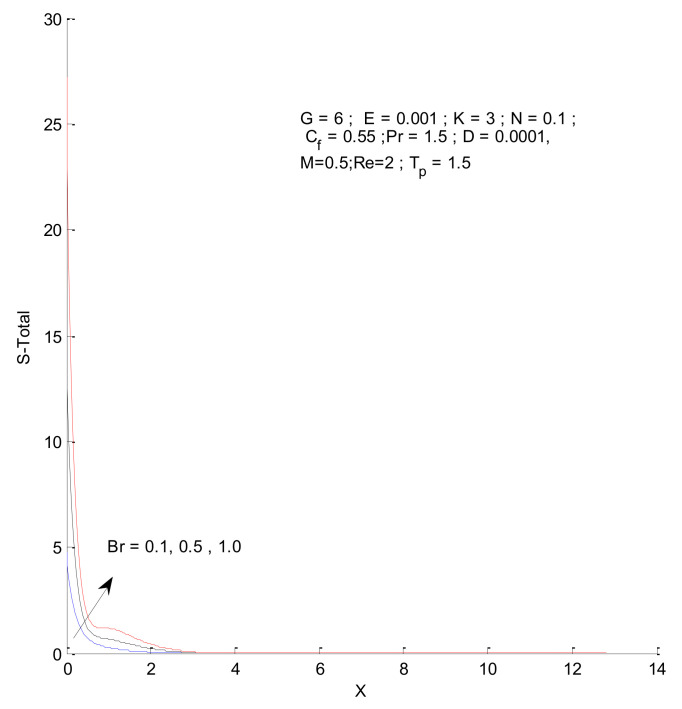
Total entropy change with respect to Br.

Figure 18, Figure 19, Figure 20 and Figure 21 illustrate the impact of the magnetic parameter on ST, SF, SM, and S-Total. As shown in these figures, the change in magnetic parameter does not have much impact on ST and SF, but slightly increases SM. The total entropy change with respect to M is marginal even though it affects SM. This is because the overall impact of SF and ST is much higher on S-Total compared to SM. Figure 22, Figure 23, Figure 24 and Figure 25 show the effect of the Brinkman number on entropy generation. Thermal entropy has a negligible effect when the Brinkman number is increased. However, fluid and magnetic entropy increase with an increase in the Brinkman number. The total entropy increases due to an increase in the Br.

**Figure 26:**
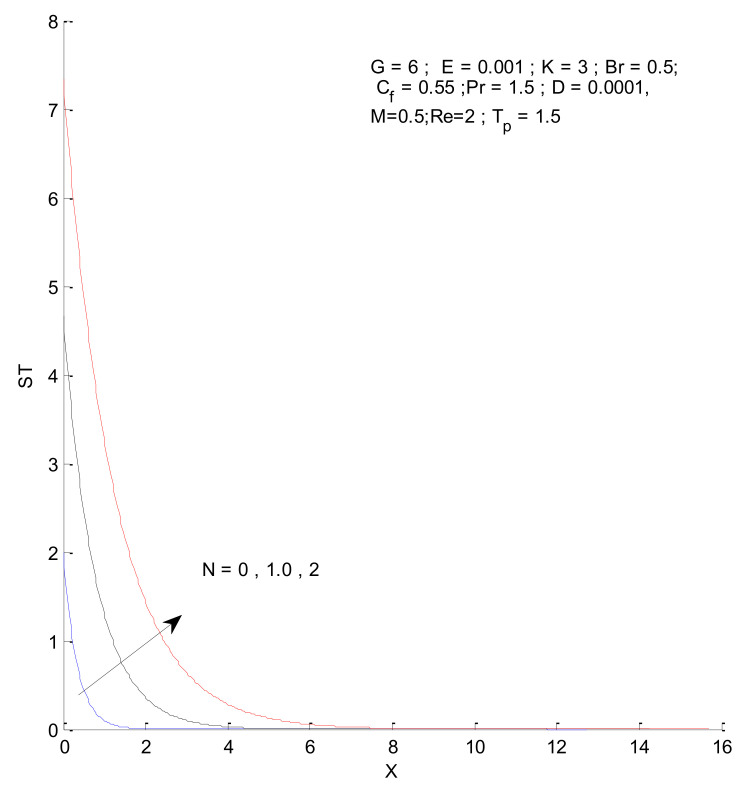
Thermal entropy change with respect to N.

**Figure 27:**
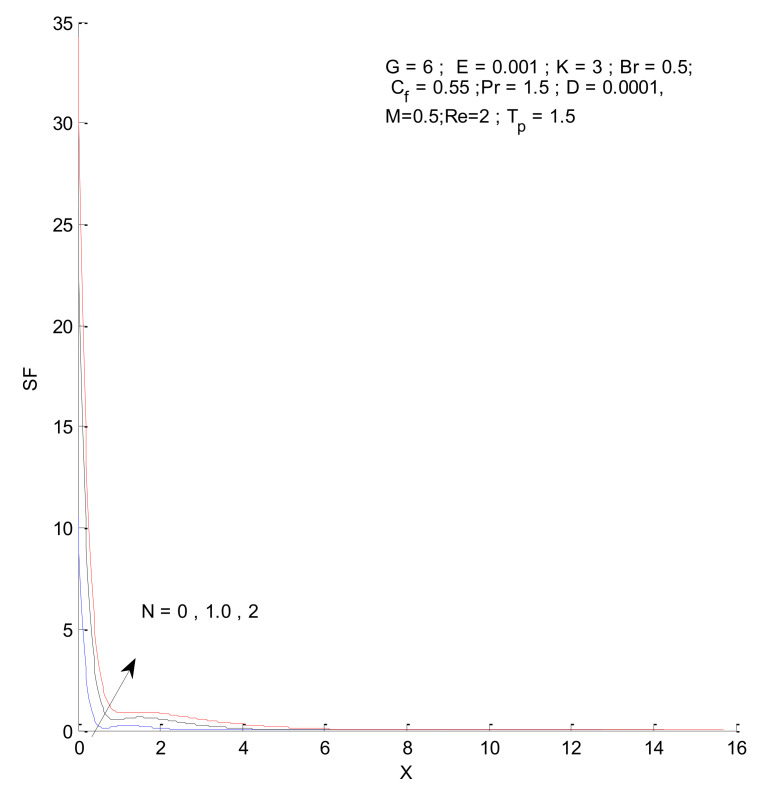
Fluid entropy change with respect to N.

**Figure 28:**
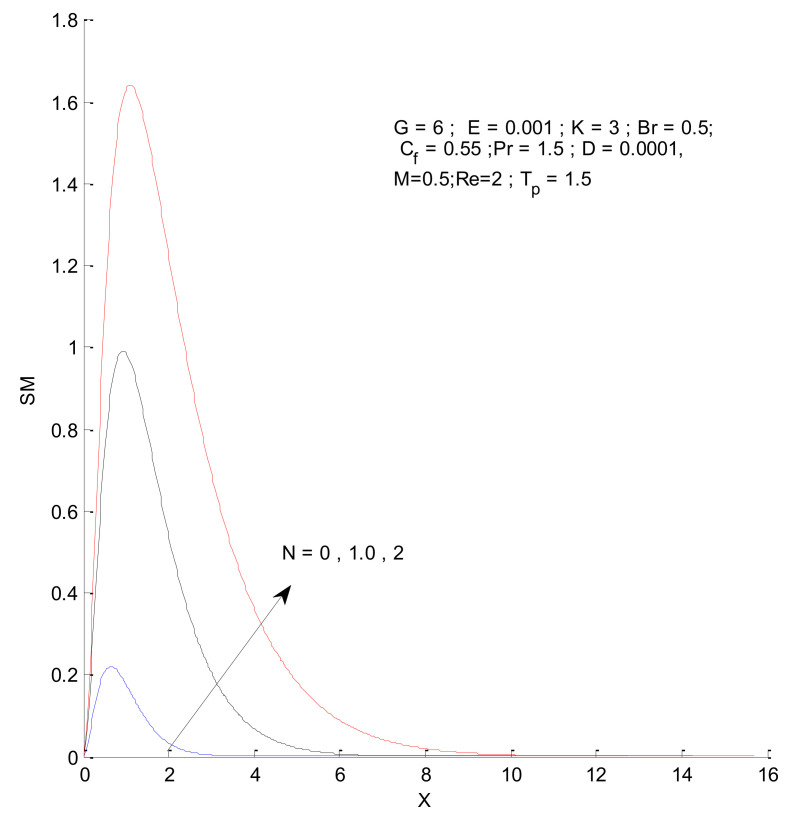
Magnetic entropy change with respect to N.

**Figure 29:**
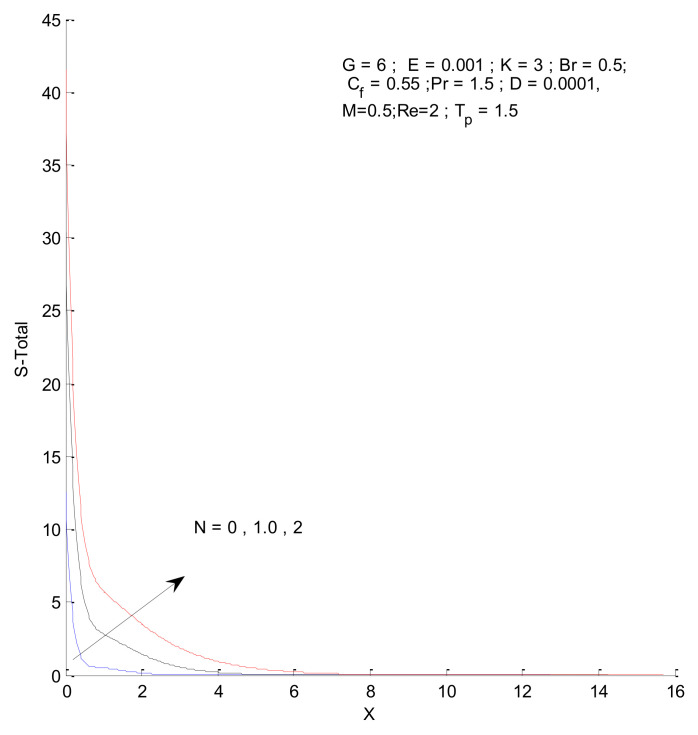
Total entropy change with respect to N.

Figure 26, Figure 27, Figure 28 and Figure 29 illustrate the effects of the radiation parameter on the entropy generation. The thermal entropy, fluid entropy, as well as magnetic entropy increase when the radiation parameter is increased. This leads to an increase in the system’s overall entropy due to an increase in the radiation effect. The total entropy of the system falls sharply close to the hot surface where the change is found to be gradual.

## 4. Conclusions

The present article investigated heat transfer behavior in a fixed porous domain adjacent to the permeable vertical plate subjected to suction velocity and magnetic effects. The analysis was carried out with respect to entropy generation due to various parameters. This work revealed the following concluding points:

- The velocity profile is affected, to a greater extent, by the Prandtl number, Grashof number, radiation parameter, magnetic parameter, and permeability parameter, whereas the impact of the Eckert number, form-drag coefficient, and viscous dissipation parameter is significantly less.
- The velocity profile decreases owing to the Prandtl number and magnetic parameter. However, a reverse trend is observed with respect to the radiation parameter, permeability parameter, and Grashof number.
- The temperature profile decreases with increasing Prandtl number, whereas it increases with increase in the radiation parameter.
- The entropy generation increases with an increase in the Reynold’s number, Brinkman number, and radiation parameter
- The fluid entropy dominates compared to thermal and magnetic entropy.
- The fluid and thermal entropy sharply decrease near the vertical plate, whereas magnetic entropy produces a different profile than the fluid and thermal entropy.

## Data Availability

None.

## Nomenclature

*B_o_*	magnetic intensity
$c_{f}$	form-drag constant
g	gravitational acceleration
G	modified Grashof number
*k*	thermal conductivity
*k**	mean absorption coefficient
K	permeability parameter
$K^{\prime }$	Permeability of porous medium
L	length
N	radiation parameter
Pr	Prandtl number
q_r_	radiation flux
T	temperature
v	vertical velocity along the plate
u_o_	suction velocity perpendicular to plate
U	dimensionless vertical velocity
x	x-coordinate in horizontal direction
X	dimensionless coordinate in horizontal direction
Pr	Prandtl number
ε	viscous dissipation parameter
M	magnetic parameter
E	Ecker number
Re	Renolds number
Br	Brinkman number
$T_{p}$	Temperature difference parameter
*Greek Symbols*	
α	thermal diffusivity
β	coefficient of thermal expansion
ρ	density
θ	dimensionless temperature
μ, *v*	coefficients of dynamic and kinematic viscosity, respectively
σ	Stephan Boltzmann constant
$\beta_{R}$	absorption coefficient
*Subscripts*	
w	wall
∞	far away condition
